# Development of a cut-off score for the growth and empowerment measure for Aboriginal and Torres Strait Islander young people (Growth and Empowerment Measure Youth)

**DOI:** 10.1177/00048674261480597

**Published:** 2026-08-28

**Authors:** Lisa Wittenhagen, Carla Meurk, Penny Dale, Scott Harden, Megan Williams, James G. Scott, Marshall Watson, Stuart Kinner, Ed Heffernan, IMHIP-Youth Cultural Governance Group

**Affiliations:** 1 Faculty of Medicine, School of Public Health, The University of Queensland, Herston, Australia; 2 Queensland Centre for Mental Health Research, Wacol, Australia; 3 Queensland Forensic Mental Health Service, Metro North Hospital and Health Service, Brisbane, Australia; 4 Forensic Child and Youth Mental Health Service, Children’s Health Queensland Hospital and Health Service, Brisbane, Australia; 5 Girra Maa Indigenous Health Discipline, Graduate School of Health, University of Technology Sydney, NSW, Australia; 6 Child Health Research Centre, The University of Queensland, South Brisbane, Australia; 7 Child and Youth Mental Health Service, Children’s Health Queensland, Brisbane, Australia; 8 Justice Health Group, Curtin University, Perth, WA, Australia; 9 Justice Health Group, Murdoch Children’s Research Institute, Melbourne, VIC, Australia; 10 School of Public Health, The University of Queensland, Brisbane, Australia; 11 Melbourne School of Population and Global Health, The University of Melbourne, VIC, Australia

## Introduction

Health research and practice have frequently failed to reflect the cultural and lived experiences of Aboriginal and Torres Strait Islander people, relying on methods and measures that do not align with individual, family, and community needs and priorities. As a result, deficit-focused and discriminatory narratives have arisen (Fogarty et al., 2018). Culturally grounded and dependable measures are vital to meet social and emotional wellbeing needs of Aboriginal and Torres Strait Islander people. Empowerment-oriented measures differ conceptually from deficit screening tools in that they focus on strengths, capacities, and individual agency rather than identifying symptoms, risks, or impairments. The Growth and Empowerment Measure Youth (GEM-Youth) was co-designed with young Aboriginal and Torres Strait Islander people to develop a culturally grounded tool that effectively evaluates the social and emotional wellbeing needs of young Aboriginal and Torres Strait Islander people and enhanced empowerment. This research letter follows up from the initial validation (Fletcher et al., 2025), providing a suggestion for a functional cut-off score for the GEM-Youth while foregrounding principles of cultural integrity and narrative understanding.

## Methods

### Study cohort and consent

A detailed account of the Indigenous Mental Health Intervention Program for youth (IMHIP-Youth) project and the development of the GEM-Youth is published elsewhere, including how it builds on work by Aboriginal and Torres Strait Islander Stolen Generations and leaders associated with the Aboriginal Family Wellbeing Program, and their development of the adult GEM (Dale et al., 2023; Fletcher et al., 2025). The IMHIP-Youth project aimed to collaboratively design, embed, and evaluate a culturally grounded model of social and emotional wellbeing care for Aboriginal and Torres Strait Islander young people in detention, in transition to the community, and in community post-detention.

Data from 88 Aboriginal and/or Torres Strait Islander young people (70 males, average age: 15.5 years, standard deviation (SD) = 1.35) who completed version 7 of the GEM-Youth and participated in phase 1 (*n* = 54) and phase 2 (*n* = 34) of the IMHIP-Youth project were extracted to establish a cut-off score for the GEM-Youth measure.

### Procedure

The GEM-Youth was developed and co-designed with a cohort of Aboriginal and Torres Strait Islander young people. The GEM-Youth is a culturally validated tool to measure social and emotional wellbeing and empowerment (Dale et al., 2023; Fletcher et al., 2025). It has two subscales: (1) ‘How I feel about myself’ (HIFAM) and (2) ‘Thinking about my everyday life’ (TAMEL). HIFAM is a 15-item Emotional Empowerment Scale with categorical answers, and TAMEL has 11 scenarios measuring functional aspects of empowerment determined by Aboriginal and Torres Strait Islander people on a categorical seven-point scale.

Responses to the HIFAM and TAMEL questions at the initial data collection contact were transformed into numerical values. Specifically, the HIFAM question scores could range between 1 and 5 and the TAMEL question scores between 1 and 7, with possible total scores for the two subscales of 75 and 77, respectively. Furthermore, a total GEM-Youth score was calculated (sum of HIFAM and TAMEL subscale scores; maximum possible score = 152). In addition, K10 scores were available for all 88 individuals. The K10 was included primarily to mirror the development of the GEM-Youth as closely as possible to that of the original GEM to support methodological consistency and comparability.

Missing data (3 cells or less) were replaced by the average score for the respective subscale item, or where responses recorded on the response sheet were ambiguous, the lower score was selected as per guidance from the Aboriginal and Torres Strait Islander Cultural Governance Group. Such data cleaning was required for six individuals.

### Analysis

Data were analysed using R version 4.3.0 (R Core Team, 2024). To determine optimal cut-off scores, a Receiver Operating Characteristic (ROC) curve was constructed using the ‘pROC’ function (Robin et al., 2011). The ROC curve is a graphical representation that evaluates the diagnostic performance of a binary classification model by illustrating the trade-off between the True Positive Rate (TPR; sensitivity) and the True Negative Rate (TNR; specificity). The ROC analysis has been employed widely in mental health research (e.g. Blake et al., 2024; Kessler et al., 2002). Total scores from the GEM-Youth were compared against a reference mainstream standard (in this case, K10 scores; K6 was used for the GEM adult), enabling the identification of a cut-off score for the newly established GEM-Youth measure. The cut-off score was determined based on Youden’s Index, which identifies the threshold that maximises the sum of sensitivity and specificity, thereby optimising overall accuracy (Youden, 1950).

Based on discussions within the IMHIP-Youth team around clinical and cultural considerations, a K10 score of 16 (indicative of individuals likely experiencing a moderate level of psychological distress; Australian Bureau of Statistics (2012)) was used as the reference threshold for binary classification (i.e. classification of total GEM-Youth scores indicating strong or weakened social and emotional wellbeing).

## Results

To evaluate the discriminative performance of the GEM-Youth, we conducted an ROC curve analysis. The model demonstrated fair ability (area under the curve (AUC) = 0.697) to distinguish between adolescents with high versus low social and emotional wellbeing. Using Youden’s Index, it showed high sensitivity (86%) but relatively low specificity (45%), indicating a tendency to prioritise detection of true positives at the expense of increased false positives. Based on this analysis, a cut-off score of 122.5 for the GEM-Youth was established (see Figure 1). Scores below this preliminary threshold may indicate young people who could benefit from further exploration of social and emotional wellbeing needs.

**Figure 1.**
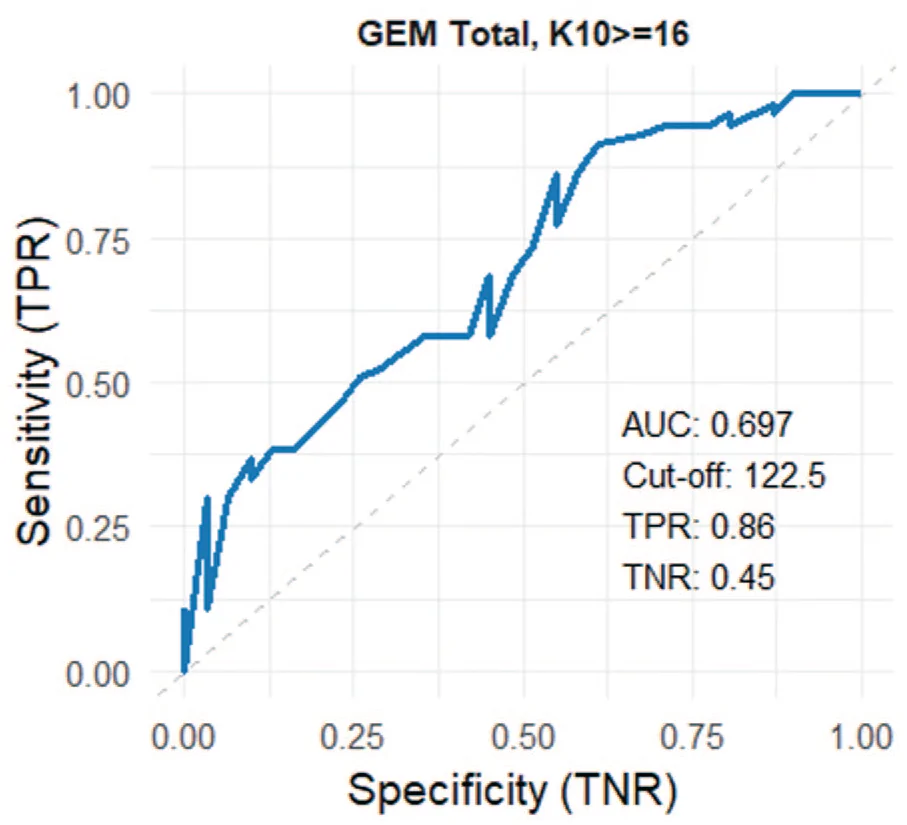
Results from the ROC curve analysis.

## Discussion

Our results contribute to the further validation of the GEM-Youth as a tool to assess social and emotional wellbeing in young Aboriginal and Torres Strait Islander people who have experienced detention. Cut-off scores are a valuable tool for identifying individuals who are more likely to require additional support, tracking response to interventions, and enabling monitoring of prevalence, outcomes, and treatment effects. Beyond individual use, aggregated (GEM-Youth) cut-off data can inform service planning, resource allocation, and programme development. Importantly, cut-off scores should not be interpreted in isolation: the social and cultural context of young people’s lives is crucial considerations. GEM-Youth cut-off scores are intended as prompts for conversation and further exploration, rather than fixed diagnostic limits. Using numbers alone can reinforce deficit-based views and may be misleading, stigmatising, or discriminatory, particularly for young people with multiple vulnerabilities. Scores must always be considered alongside the young person’s context, rights, needs, and story, particularly those gathered through yarning and qualitative research.

### Limitations

The sample used to validate the GEM-Youth and determine its preliminary cut-off score was relatively small, and its development has occurred exclusively within detention settings. To enhance the GEM-Youth’s applicability and robustness, further research must examine its appropriateness and performance across a range of individual, family, and community contexts. A more suitable sample size for validation and for generating a stable cut-off score would require a significantly larger cohort. Importantly, with larger and more diverse samples, cut-off scores can be defined in a way that is intrinsic to the tool itself (such as computing *z*-scores), rather than relying on external normative mainstream standards such as the K10. This approach would enhance the validity, cultural relevance, and practical utility of the tool and its role in supporting empowerment and the social and emotional wellbeing of young Aboriginal and Torres Strait Islander people.
